# Risk of Cardiac Lesion with Chronic and Acute Use of Loperamide—An Integrative Review

**DOI:** 10.3390/jcdd9120431

**Published:** 2022-12-02

**Authors:** Bruna Cremonezi Lammoglia, Gabriela Hasselmann, Marcelo Pires-Oliveira, Lucas Antonio Duarte Nicolau, Jand Venes Rolim Medeiros, Fernando Sabia Tallo, Murched Omar Taha, Rildo Yamaguti Lima, Afonso Caricati-Neto, Francisco Sandro Menezes-Rodrigues

**Affiliations:** 1 School of Medicine, Universidade Santo Amaro (UNISA), São Paulo 04829-300, SP, Brazil; 2 School of Medicine, União Metropolitana de Educação e Cultura, Lauro de Freitas 42700-000, BA, Brazil; 3 Laboratory of Pharmacology of Inflammation and Gastrointestinal Disorders (LAFIDG), Universidade Federal do Delta do Parnaíba (UFDPar), Parnaíba 64202-020, PI, Brazil; 4 Department of Urgency and Emergency Care, Universidade Federal de São Paulo (UNIFESP), São Paulo 04023-062, SP, Brazil; 5 Department of Surgery, Universidade Federal de São Paulo (UNIFESP), São Paulo 04023-062, SP, Brazil; 6 Postgraduate Program in Cardiology, Universidade Federal de São Paulo (UNIFESP), São Paulo 04023-062, SP, Brazil; 7 Department of Pharmacology, Universidade Federal de São Paulo (UNIFESP), São Paulo 04023-062, SP, Brazil

**Keywords:** loperamide, opioid-related disorders, cardiotoxicity, long QT syndrome, torsades de pointes

## Abstract

Loperamide is a synthetic opioid commonly used as an antidiarrheal due to its activation of u-opioid receptors in the myenteric plexus. In therapeutic doses, it inhibits peristalsis and has anti-secretory and anti-motility effects, until metabolized by intestinal and hepatic CYP3A4 and CYP2C8 into inactive metabolites. Furthermore, loperamide also inhibits L-type voltage-gated calcium (Ca^2+^) channels, increases action potential duration, and can induce arrhythmias and even cardiotoxicity, particularly when taken in extremely high doses. Thus, the aim of this study was to perform an integrative review of the available evidence in the recent literature on the cardiac risks of acute and chronic use of loperamide. In electrocardiogram (ECG) analysis, the most common finding was QTc prolongation in 27 cases, followed by QRS prolongation, first-degree atrioventricular (AV) block, torsades de pointes, ventricular tachycardia, and right bundle branch block. As for the symptoms encountered, syncope, weakness, palpitations, lightheadedness, shortness of breath, nausea, vomiting, bradycardia, and cardiac arrest were the most common. Loperamide can inhibit hERG voltage-gated potassium (K^+^) channels (Kv11.1), leading to the prolongation of repolarization, QTc interval prolongation, and increased risk of torsades de pointes. In addition, loperamide can inhibit voltage-gated sodium (Na^+^) channels (Nav1.5), impairing electrical cardiac conduction and potentiating QRS interval widening. Therefore, QTc prolongation, torsades de pointes, and other ECG alterations are of particular concern regarding loperamide toxicity, particularly when overdosed.

## 1. Introduction

Loperamide is a synthetic opioid commonly used as an antidiarrheal. Its main therapeutic effects result from the activation of µ-opioid receptors in the myenteric plexus, until it is metabolized by intestinal and hepatic CYP3A4 and CYP2C8 into inactive metabolites. In therapeutic doses, it also inhibits peristalsis and has anti-secretory and anti-motility effects [1]. Loperamide is a substrate of P-glycoprotein-mediated efflux, which explains its low net penetration of the blood–brain barrier. Consequently, loperamide does not produce analgesia, euphoria, or respiratory depression at doses ≤ 16 mg/day, and it is classified as a drug with low abuse potential [2]; however, when ingested in high doses, loperamide crosses the blood–brain barrier due to P-Glycoprotein saturation and may produce euphoria and analgesia, which can lead to drug abuse [1].

It was previously shown that loperamide is a blocker of cardiac hERG voltage-gated potassium (K^+^) channels (Kv11.1) and Nav1.5. Blockade of cardiac Kv11.1 channels is commonly associated with drug-induced QT interval prolongation, whereas the blockade of cardiac Nav1.5 channels leads to QRS interval widening. In addition, loperamide also blocks L-type voltage-activated Ca^2+^ channels, increases action potential duration, and can induce other arrhythmias [3] and even cardiotoxicity in extremely high doses [2].

In 2016, the U.S. Food and Drug Administration (FDA) warned about the risk of QT interval prolongation, torsades de pointes arrhythmia, and cardiac arrest at higher-than-recommended doses [1]. Since then, new reports of potential cardiac complications of loperamide use have appeared in the scientific literature. Thus, the aim of this study was to analyze the available evidence in the recent literature on the cardiac risks of the acute and chronic use of loperamide.

## 2. Materials and Methods

The study is an integrative review of the literature performed according to Toronto, C. and Remington, R. (2020) about the risk of arrhythmias and cardiac lesions with the acute or chronic use of loperamide [4]. The research was carried out in the PubMed, Embase, Scopus, and BVS databases. Articles in Portuguese, English, or Spanish between 2018 and 2022 were included. The descriptors searched were ‘loperamide’, ’ECG’, ‘EKG’, ‘loperamide abuse’, and ‘cardiotoxicity’. A total of 190 research papers were found, of which 118 were non-duplicates. After evaluation by title, 59 potentially relevant papers were considered. Of these, after a new evaluation of the abstracts, 13 could not be obtained in full and were excluded, and 46 were considered. The 46 papers were read in full, and, of these, 29 relevant papers were selected to compose the final sample for this study.

## 3. Eligibility Criteria

Only original case reports of cardiac toxicity or cardiac arrhythmias after loperamide abuse, overuse, or chronic use were included. An irrelevant study was defined as a non-case report, such as commentaries or letters to the editor.

Articles that had no mention in their title of the use of loperamide or mentioned abuse of other opioids and drugs in general, management of diarrhea with no mention of loperamide abuse, and clinical studies in animals were excluded.

During the selection by abstract, animal studies, letters to the editor, case reports in which more than one type of opioid was abused in addition to loperamide, articles that only mentioned loperamide but not the abuse of the drug, and articles that were not found in the media were discarded. We included articles that mentioned management after loperamide abuse, case reports of loperamide abuse in humans, loperamide cardiotoxicity, and syndromes diagnosed after loperamide abuse.

Finally, the selection of full articles included only case reports that mentioned changes in electrocardiogram after abuse of loperamide in humans; articles in which abuse of more than one type of opioid was reported were excluded. Previous cardiovascular disease or other comorbidities were not considered as exclusion or inclusion criteria.

## 4. Selection Process

The study was conducted by two reviewers who analyzed the initial sample separately, applying the same filters and inclusion and exclusion criteria, and both reached the same final sample.

For the analysis, Microsoft Excel was used to organize the articles to generate a table from the databases by applying the filters: selected descriptors and articles published in the last 5 years. All selection was performed manually by the reviewers without associated tools.

**Figure 1:**
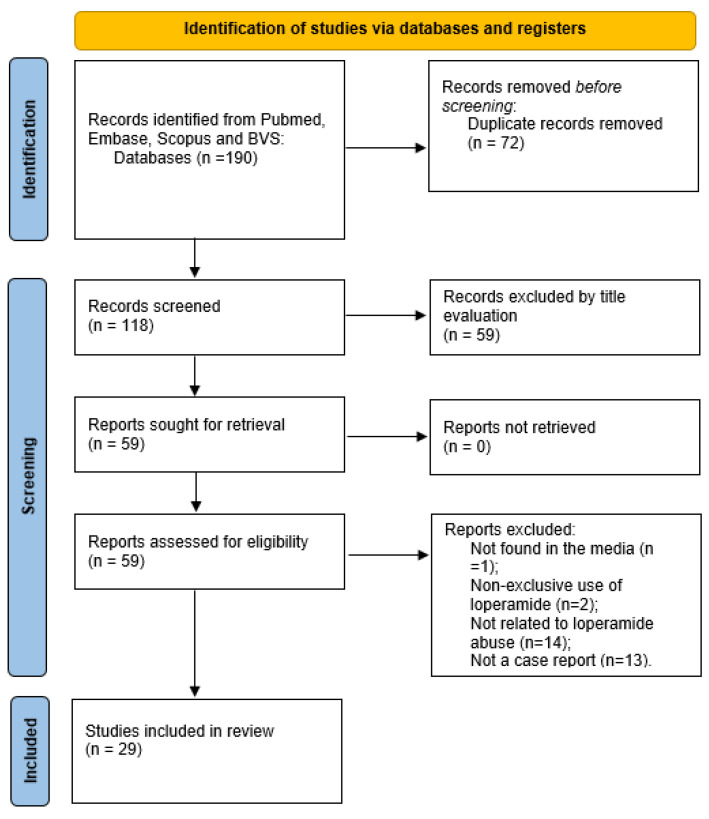
Flow chart of the selection process for case reports of cardiac lesions with the acute or chronic use of loperamide.

Provided the sample of this study, we extracted the author, year of publication, age, dose of ingested loperamide, symptoms/signs, ECG results, treatment, and clinical outcome (Figure 1).

## 5. Results

This article reviewed a total of 29 cases of intentional abuse or non-intentional misuse of loperamide. Fourteen patients (48%) were male and fifteen (52%) were female. Their ages ranged from 14 to 49 years old; the mean age was 31.5 years (30 years among males; 33 years among females).

Table 1 details all male cases; the dose of ingested loperamide ranged from 20 to 600 mg; the mean dose was 181.5 mg. One case did not report a loperamide dose. The most common symptom was syncope in six cases (43%); weakness was observed in 23% of patients; other common symptoms were cardiac arrest, lightheadedness, nausea and vomiting, shortness of breath and abdominal pain (15% of cases); palpitations, dizziness, and tonic–clonic seizures (8% of cases).

Most ECG analyses of males (93%) showed QTc and QRS elevation. QTc ranged from 500 to 800 ms; the mean interval was 632 ms. QRS was included in eight cases, in which it was found to be elevated, between 136 and 170 ms. In two cases, ST elevation was seen, consistent with type 1 Brugada patterns. Other alterations included AV block with sinus bradycardia; ventricular arrhythmias and ventricular tachycardia; and torsades de pointes (each in 15% of cases).

In 50% of cases, ECGs returned to normal in 3–5 days after treatment. A diagnosis of Brugada syndrome was made in two cases, one of which was secondary to loperamide abuse.

Table 2 details female cases. The dose of ingested loperamide ranged from 12 mg to 600 mg; the mean dose was 205.56 mg, and one case did not report the loperamide dose ingested. The most common symptoms were syncope (44%) and palpitations (25%). Other symptoms included: bradycardia (19%); dyspnea (19%); arrhythmia (13%); generalized weakness (13%); and, less commonly, hypoxia; hypertension; atypical chest pain; shortness of breath; hypotension; tachycardia; bradypnea; hypoglycemia; light-headedness; and loss of consciousness.

All ECGs of female patients showed QTc elevation in the 425–831 ms range; the mean interval was 621 ms. QRS was included in six cases, in which it was found to be elevated, between 142 and 270 ms. In 25% of cases, there was first-degree AV block; in 19%, torsades de pointes; in 13%, prolonged PR interval; and in 13%, ST elevation. In seven cases, ECGs returned to normal in 1–3 days after the treatment.

Considering all cases in both tables, 13 cases (50%) ingested more than 200 mg of loperamide, and 13 cases (50%) ingested less than 200 mg; one case reported ingestion of 96 to 320 mg, and two cases (6.9%) did not report a loperamide dose. The most common ECG alterations were QTc prolongation in 27 cases (93%), followed by QRS prolongation in 12 cases (41%); first-degree AV block in 6 cases (20%); torsades de pointes in 5 cases (17%); ventricular tachycardia and right bundle branch block in 3 cases each (10%); idioventricular rhythm, prolonged PR, and inverted T waves, each in 2 cases (6.8%); and tachycardia, sinus tachycardia, S3 gallop, and ventricular fibrillation in 1 case each (3.4%).

As for the symptoms encountered, there were 13 cases (44%) with syncope, 5 cases (17%) with weakness, 5 cases (17%) with palpitations, 4 cases (13%) with lightheadedness, and 4 cases (13%) with shortness of breath. Nausea, vomiting, and bradycardia were present in three cases (10%). Cardiac arrest was present in two cases (6.8%), as were tachycardia, arrhythmia, and abdominal pain. Diaphoresis, constipation, hypothermia, bradypnea, hypoglycemia, hypotension, chest pain, hypertension, dyspnea, itching, confusion, loss of consciousness, gastrointestinal symptoms, hypoxia, and lethargy were present in one case each (3.4%), and one case occurred in which no symptom was recorded.

The most common treatments in both tables were magnesium sulfate, given in 15 cases (52%) and sodium bicarbonate, given in 8 cases (28%). Isoproterenol was utilized in five cases (17%). Lidocaine was administered in three cases (10%) and amiodarone in four cases (14%). Betamethasone, furosemide, nitroglycerin, NIPPV, potassium replacement, supportive care, metoprolol, naloxone, N-acetylcysteine infusion, dextrose, atropine, dopamine, norepinephrine, hydration, cardiac monitor, inotropic support, loop recorder, cardioconversion, acetaminophen, morphine, lorazepam, and ondansetron were each used in only one case.

Analyzing the outcomes, in 16 cases (55%), the ECG returned to normal; 4 cases (13%) had torsades de pointes; in 3 cases (13%), isoproterenol was used; 3 cases (13%) were put in a temporary pacemaker; 2 cases (6.8%) cases occurred with Brugada syndrome; and in 2 cases (6.8%), electrical cardioversions were performed. A holter was placed in one case (3.4%); buprenorphine–naloxone or buprenorphine was also administered in one case (3.4%) each. Hypoxia-related brain injury was reported in one case (3.4%), as well as resolution of tachycardia and bradycardia, cardiopulmonary resuscitation, and monitoring for 72 h. In other cases, there were: recommended follow-ups with a cardiologist (2 cases; 6.8%), a psychiatrist (1 case; 3.4%), and genetic testing (1 case; 3.4%). There was also a case in which the patient developed ventricular tachycardia and ventricular fibrillation.

## 6. Discussion

The mechanism of loperamide’s cardiotoxicity is currently unknown. The most likely related mechanism is the inhibition of voltage-gated sodium and potassium channels in cardiac cells. Nav1.5 channels are responsible for action potential amplitude, impulse conduction velocity, and successful propagation. Thus, since loperamide also blocks Nav1.5 channels, which may result in the impairment of electrical cardiac conduction, leading to the potentiation of QRS interval widening and Brugada patterns [5,6,7,10,19,32,33,34]. The blockade of Kv11.1 channels by loperamide results in the prolongation of repolarization that produces QTc interval prolongation and increases the risk of torsades de pointes. Repolarization delay leads to early after-depolarization, heterogeneous myocardial repolarization, and torsades de pointes caused by the inhibition of the delayed rectifier potassium current. QTc prolongation is a significant concern regarding loperamide toxicity [7,9,19].

Previous reports of loperamide toxicity show a broadening of the QRS complex and the prolongation of the QT interval in relation to the onset of cardiac arrhythmias such as tachycardias, polymorphic ventricular tachycardia, and sudden cardiac death. QRS and QTc prolongation was the most common cardiac alteration found in the study, followed by first-degree AV block with sinus bradycardia, ST elevation, torsades de pointes, and Brugada pattern [19,35]. Teigeler et al., 2019 [9] published the ECG demonstrated the polymorphic ventricular tachycardia, with prolonged QTc, after the abuse of loperamide.

Initially, patients should be evaluated for any reversible risk factors for QTc prolongation, such as electrolyte abnormalities such as hypokalemia and hypomagnesemia. For the treatment of loperamide-induced cardiotoxicity, each case must be individualized with its due comorbidities and changes; however, in an analysis of the 29 articles used as a basis for the work, the main treatment options can be listed according to their greater efficiency (without dosage specified in the articles): the IV infusion of magnesium sulfate, the IV infusion of sodium bicarbonate, IV lidocaine, IV amiodarone, and atropine. Torsades secondary to loperamide toxicity may not respond well to therapies with magnesium or sodium bicarbonate; thus, other agents such as atropine, amiodarone, lidocaine, and metoprolol might be necessary. Treatment may also require cardiac pacing, electrical cardioversion, or IV isoproterenol in some cases to achieve heart rate overdrive for arrhythmia control. When associated ventricular dysrhythmias occur, treatment with magnesium sulfate and sodium bicarbonate has been successful [19,33].

Furthermore, processes of decontamination, increased elimination, and the use of an antidote and supportive care might be considered in the treatment of loperamide-induced cardiotoxicity. Activated charcoal can be used for loperamide adsorption and decontamination, as it can considerably reduce loperamide concentrations due to reduced gastrointestinal smooth muscle motility. On the other hand, as of now, there are no effective evidence-based strategies to promote the elimination of loperamide [16]. Loperamide-induced cardiotoxicity is at least partially caused by the blockade of sodium channels. This likely explains why the administration of IV sodium bicarbonate may help attenuate arrhythmogenic effects of loperamide, such as QTc interval prolongation. Nevertheless, the use of sodium bicarbonate may itself cause concerning changes in serum potassium and magnesium concentrations.

There are no specific recommendations regarding patient life support, but general actions to be considered as possible supportive treatments for loperamide toxicity include defibrillation; the intravenous administration of magnesium sulfate, amiodarone, or lidocaine; and excessive stimulation. The half-life of loperamide is relatively long, between 9 and 30 h, and the electrical disturbances that lead to the onset of cardiac arrhythmias due to the actions of loperamide persist for several days [16]. The data on the use of magnesium sulfate in the treatment of cardiac arrhythmias are conflicting, as there is evidence to suggest that it is unlikely to result in decreased mortality when introduced either early or later in patients with fibrinolytic treatment or even in high doses, i.e., greater than 75 mmol. In addition, there may be an increased incidence of cardiovascular disorders, such as bradycardia, hypotension, and flushing [36,37]. However, there is also evidence to show that magnesium administration can reduce the incidence of major ventricular arrhythmias, such as ventricular fibrillation and ventricular tachycardia.

## 7. Conclusions

When overdosed, loperamide blocks cardiac sodium and potassium channels, resulting in a variety of cardiac rhythm abnormalities, such as QTc and QRS prolongation, first-degree AV block, torsades de pointes, ventricular tachycardia, and right bundle branch block.

## Figures and Tables

**Table 1:** Male cases.

Author	Age	Dose of Ingested Loperamide	Electrocardiogram (ECG)	Symptoms/Signs	Treatment	Clinical Outcome
Sun C. et al., 2018 [5]	49	200 to 400 mg	Corrected QT (QTc) of 644 ms and QRS of 170 ms	-	IV infusion of magnesium	6 months before the ECG showed a QTc interval of 429 ms and normal ST segments
Stefek B. et al., 2018 [6]	14	40 mg nightly	ECG revealing anterior precordial ST segment elevation in leads V1–V3 with a “coved” appearance consistent with a type 1 Brugada pattern QRS duration was 160 ms, and QTc interval was 568 ms	Nausea, vomiting, diaphoresis, dizziness and weakness	Magnesium sulfate, ondansetron, sodium bicarbonate infusion, pantoprazole, lorazepam, morphine, and acetaminophen	A diagnosis of Brugada syndrome was made
Simon M. and Rague J., 2021 [7]	22	600 mg	ECG showed a prolonged QTcR interval of 667 ms, QRS of 136 ms, with ST elevations in V1–V3 with a “coved” appearance followed by a negative T wave consistent with a Type 1 Brugada pattern	Multiple witnessed syncopal episodes	IV magnesium sulfate	The patient receieved an isoproterenol infusion
Jablonski S. et al., 2019 [8]	33	>100 mg	ECG showed sinus bradycardia, first-degree atrioventricular (AV) block, QRS interval of 192 ms and QTc of 615 ms	Two witnessed cardiac arrests, both with torsades de pointes	Magnesium and cardioversion followed by temporary venous pacemaker placement	ECG and echocardiogram normalized on day 3 of admission with no further dysrhythmia
Teigeler T. et al., 2019 [9]	39	40–60 mg twice daily	ECG revealed an idioventricular rhythm at a rate of 34 bpm, QRS duration of 154 ms, and a QTc of 444 ms	Lightheadedness and presyncope	-	Bradycardia resolved. ECG normalized after 5 days
Kohli U. et al., 2019 [10]	26	300 mg	ECG showed markedly prolonged QTc and Brugada type 1 changes in leads V1 and V2. QTc-prolonged (>500 ms)	Syncope	A loop recorder was placed	Discharged home with a life vest
Escobedo Y. et al., 2020 [11]	30	140 mg	EMS found refractory torsades de pointes, a normal sinus mechanism with a normal QTc that progressively increased to 800 ms	Witnessed cardiac arrest	Veno-arterial extracorporeal membrane oxygenation (VA ECMO) and inotropic support	Echocardiogram revealed normalized LVEF on discharge
Atoot A. et al., 2020 [12]	28	280 mg	Echocardiogram demonstrated no regional wall motion abnormalities, and ST-segment elevation in V1–V3 leads	Excruciating abdominal pain	-	Transient Brugada pattern secondary to loperamide abuse
Ali M. et al., 2020 [1]	31	400 mg	ECG revealed a widened ventricular arrhythmia, short runs (<3 s) of ventricular tachycardia, and prolonged QTc of 663 ms	Shortness of breath and weakness	IV magnesium and bicarbonate drip	4 days after hospitalization, the ECG had improved back to normal sinus rhythm with prolonged QTc
Palkar P. and Kothari D., 2018 [13]	32	>100 mg	ECG showed right bundle branch block, bradycardia with a heart rate of 51 bpm, and normal QTc	Syncopal episode, nausea, vomiting, constipation, and abdominal cramps	Cardiac monitor with normal saline intravenous for hydration	Discharge with a Holter monitor
Modi V. et al., 2021 [14]	28	200 mg	ECG showed widened QRS and prolonged QT interval. After treatment with a bicarbonate drip, the patient developed an episode of polymorphic ventricular tachycardia	Weakness, difficulty taking deep breaths, and mild lightheadedness	Sodium bicarbonate	Temporary pacemaker
Larsen T. et al., 2018 [15]	32	200 mg	ECG showed sinus tachycardia at 101 bpm, first-degree AV block, nonspecific intraventricular conduction delay, and prolonged QT interval	Severe palpitations and syncope	Intravenous magnesium and supportive care	After 5 days PR interval, QRS duration, and normalized QT interval and resolved VT
Sheikh S. and Baig M.A., 2019 [16]	30	-	ECG showed prolonged QT and QTc intervals of 600 ms. Cardiac monitoring showed torsades de pointes with hemodynamic instability	Generalized tonic–clonic seizures	Intubation, IV magnesium and IV amiodarone	The patient needed cardiopulmonary resuscitation. Temporary pacemaker
Stone B. et al., 2021 [17]	41	400 mg	ECG revealed sinus bradycardia with prolonged PR, QRS, and QTc intervals at 214, 148, and 678 ms, respectively	Syncope	Intensive care unit on norepinephrine, dopamine, and isoproterenol	Started on buprenorphine–naloxone and discharged. He stabilized on day three (QTc normal)

**Table 2:** Female cases.

Author	Age	Dose of Ingested Loperamide	Electrocardiogram (ECG)	Symptoms/Signs	Treatment	Clinical Outcome
Parker M.B. et al., 2019 [18]	36	400–600 mg	ECG revealed torsades de pointes, QRS 170 ms; QTc 831 ms	Bradycardia, hypothermia, bradypnea, and hypoglycemia	VA-ECMO, sodium bicarbonate infusion, N-acetylcysteine infusion, dextrose, atropine, and magnesium sulfate	ECG revealed a narrow QRS complex with normalized QTc to 410 ms
Isang E. et al., 2021 [19]	34	96 mg	ECG showed first-degree AV block, with a heart rate of 86 bpm and a prolonged QTc of 560 ms	Gastrointestinal symptoms, lethargy, hypoxia, and bradycardia	Naloxone, intravenous magnesium, bicarbonate infusion, and isoproterenol	Torsades de pointes and was successfully defibrillated with 120 J
Kapaganti S. et al., 2020 [20]	38	Took an unknown amount of loperamide	Prolonged QTc of >600 ms	Unresponsive	Lidocaine drip, electrolyte repletion	A repeat EKG showed that QTc had shortened to less than 500 ms within 24 hhypoxic brain injury
Cicci J.D. et al., 2019 [2]	23	160 mg	ECG showed pulseless ventricular fibrillation with a QTc of 554 ms and a cove-like ST-elevation pattern. Episodes of torsades de pointes	Arrhythmias	Multiple defibrillators and IV lidocaine. Mechanical circulatory support, IV magnesium	Torsades de pointes (TdP) (QTc 613 ms) requiring three electrical cardioversions
Whittaker G. and Newman J., 2021 [21]	30	100 mg	ECG showed a prolonged QTc ranging between 553 and 567 ms. First-degree AV block and right bundle branch block were also present	Syncope, palpitations, light-headedness, and loss of consciousness	Lidocaine IV	Switch the loperamide to buprenorphine patches. Cardiologist and psychiatrist evaluation
Rawala M.S., 2020 [22]	33	200 mg	ECG showed QTc of 647 ms and wide complex tachycardia	Syncope	IV amiodarone	Discharged on mexiletine with normal QTc
Vera De J. et al., 2021 [23]	29	600 mg	ECG showed polymorphic ventricular tachycardia with prolonged QTc of 669 ms. Recurrent episodes of ventricular tachycardia that degenerated into torsades de pointes	Altered, confused, tachycardic, and hypotensive	IV magnesium sulfate and sodium bicarbonate	Multiple cardioversions, admission to the cardiac intensive care unit and Isoproterenol
Gaines H. et al., 2020 [24]	28	400 mg	ECG showed junctional rhythm and QTc of 557 ms	Bradycardia and syncope	IV magnesium, defibrillation and isoproterenol	Isoproterenol for 10 days
Rojas S.F. et al., 2018 [25]	48	12 a 18 mg/day	ECG mimicked type 1 Brugada pattern on leads V1–V2 and showed right axis deviation (RAD), first-degree AV block with PR interval 339 ms, and right bundle branch block with QRS duration of 270 ms and QTc of 578 ms	Syncope, palpitations, and generalized weakness	Supportive care	Monitoring by 72 h until electrocardiographic abnormalities were resolved
Idris A. and Kaye K., 2018 [26]	33	140 mg	ECG showed sinus rhythm with first-degree AV block, normal QRS, but prolonged QT/QTc intervals of 586–724 ms	Acute onset of shortness of breath, generalized weakness, and tingling over her entire body	IV infusion of sodium bicarbonate	ECG after bicarbonate showed improved QT/QTc, up to 466/531 ms at 24 h. The patient remained asymptomatic and was discharged. Ten days after discharge, QT/QTc intervals were 426/460 ms
Khan N. et al., 2021 [27]	25	400 mg	QT prolongation and giant inverted T waves in V4–V6	Palpitations and arrhythmia	Metoprolol succinate	Follow-up visit with a cardiologist and genetic testing
Zaman M.O., et al., 2018 [28]	25	30 mg daily	ECG showed non-specific ventricular rhythm with markedly increased QRS and QT interval and S3 gallop	Syncope	Amiodarone, magnesium, and sodium bicarbonate	Discontinuation of loperamide use
Wang A. et al., 2019 [29]	33	40 mg daily	ECG showed an accelerated idioventricular rhythm, widened QRS (183 ms) and prolonged QTc (700 ms)	Hypertension and dyspnea	Betamethasone, furosemide, nitroglycerin, and nasal intermittent positive pressure ventilation (NIPPV)	Medical treatment and urgent cesarean section. After 5 days in hospital, her QTc stabilized
Sapra A. et al., 2019 [30]	32	400 mg	ECG showed persistently prolonged QT interval, and nonspecific ST changes were noted	Atypical chest pain, palpitations, syncope, episode of polymorphic ventricular tachycardia, and shortness of breath	IV amiodarone and magnesium	Admission to the intensive care unit, the QT interval stabilized, and implantable cardiac defibrillator (ICD) therapy
Myllymäki L. et al., 2020 [31]	40	96 to 320 mg	ECG showed irregular heartbeat, widened QRS complex of 142 ms, and QTc of 465 ms	Syncope	IV magnesium, potassium replacement	Temporary pacemaker was inserted prophylactically, and torsades de pointes

## Data Availability

The original contributions presented in the study are included in the article, further inquiries can be directed to the corresponding authors.
